# Supplementary material for: Trauma-informed approaches to primary and community mental health care: protocol for a mixed-methods systematic review
Source: BMJ Open. 2021 Feb 18;11(2):e042112. doi: 10.1136/bmjopen-2020-042112 (PMC7896604; doi:10.1136/bmjopen-2020-042112)
Supplement: Supplementary data [file bmjopen-2020-042112supp001.pdf]

Appendix 1: Preferred Reporting Items for Systematic review and Meta-Analysis Protocols (PRISMA-P) checklist (Shamseer et al. 2015)[29]

Section and topic	Item No	Checklist item	Reported on Page #
**Administrative Information**			
**Title**			
Identification	1a	Identify the report as a protocol of a systematic review	1 (Title)
Update	1b	If the protocol is for an update of a previous systematic review, identify as such	NA
Registration	2	If registered, provide the name of the registry (e.g., PROSPERO) and registration number in the Abstract	4 (Abstract)
**Authors**			
Contact	3a	Provide name, institutional affiliation, and e-mail address of all protocol authors; provide physical mailing address of corresponding author	1-2
Contributions	3b	Describe contributions of protocol authors and identify the guarantor of the review	19-20 (Author Contributions)
Amendments	4	If the protocol represents an amendment of a previously completed or published protocol, identify as such and list changes; otherwise, state plan for documenting important protocol amendments	NA
**Support**			
Sources	5a	Indicate sources of financial or other support for the review	20 (Funding statement)
Sponsor	5b	Provide name for the review funder and/or sponsor	20 (Funding statement)
Role of sponsor/funder	5c	Describe roles of funder(s), sponsor(s), and/or institution(s), if any, in developing the protocol	NA
**Introduction**			
Rationale	6	Describe the rationale for the review in the context of what is already known	5-8 (Introduction)
Objectives	7	Provide an explicit statement of the question(s) the review will address with reference to participants, interventions, comparators, and outcomes (PICO)	9, 9-12 (PICO)
**Methods**			
Eligibility criteria	8	Specify the study characteristics (e.g., PICO, study design, setting, time frame) and report characteristics (e.g., years considered, language, publication status) to be used as criteria for eligibility for the review	9-12 (eligibility criteria)

1

Information sources	9	Describe all intended information sources (e.g., electronic databases, contact with study authors, trial registers, or other grey literature sources) with planned dates of coverage	12-13 (search strategy)
Search strategy	10	Present draft of search strategy to be used for at least one electronic database, including planned limits, such that it could be repeated	Appendix 2 (Supplementary file)
Study records			
Data management	11a	Describe the mechanism(s) that will be used to manage records and data throughout the review	13 (Screening of studies, paragraph 1)
Selection process	11b	State the process that will be used for selecting studies (e.g., two independent reviewers) through each phase of the review (i.e., screening, eligibility, and inclusion in meta-analysis)	13 (Screening of studies, paragraph 1)<br>14 (Data extraction, paragraph 2)
Data collection process	11c	Describe planned method of extracting data from reports (e.g., piloting forms, done independently, in duplicate), any processes for obtaining and confirming data from investigators	14 (Data extraction, paragraph 2)
Data items	12	List and define all variables for which data will be sought (e.g., PICO items, funding sources), any pre-planned data assumptions and simplifications	14 and 15 (Data extraction)
Outcomes and prioritization	13	List and define all outcomes for which data will be sought, including prioritization of main and additional outcomes, with rationale	Figure 1 (Supplementary file)
Risk of bias in individual studies	14	Describe anticipated methods for assessing risk of bias of individual studies, including whether this will be done at the outcome or study level, or both; state how this information will be used in data synthesis	15 (Quality appraisal)
Data Synthesis	15a	Describe criteria under which study data will be quantitatively synthesized	NA
	15b	If data are appropriate for quantitative synthesis, describe planned summary measures, methods of handling data, and methods of combining data from studies, including any planned exploration of consistency (e.g., I^2, Kendall's tau)	NA
	15c	Describe any proposed additional analyses (e.g., sensitivity or subgroup analyses, meta-regression)	NA
	15d	If quantitative synthesis is not appropriate, describe the type of summary planned	15 (Synthesis)
Meta-bias(es)	16	Specify any planned assessment of meta-bias(es) (e.g., publication bias across studies, selective reporting within studies)	NA
Confidence in cumulative evidence	17	Describe how the strength of the body of evidence will be assessed (e.g., GRADE)	NA

*NA- Not Applicable

Dawson S, *et al. BMJ Open* 2021; 11:e042112. doi: 10.1136/bmjopen-2020-042112
