# Supplementary material for: Trauma-informed approaches to primary and community mental health care: protocol for a mixed-methods systematic review
Source: BMJ Open. 2021 Feb 18;11(2):e042112. doi: 10.1136/bmjopen-2020-042112 (PMC7896604; doi:10.1136/bmjopen-2020-042112)
Supplement: Supplementary data [file bmjopen-2020-042112supp002.pdf]

Appendix 2: Example search strategy

Database(s): **Ovid MEDLINE(R)** 1946 to present

Search Strategy:

#	Searches	Results
1	(trauma inform* or trauma-inform* or trauma focus* or trauma-focus* or trauma responsive* or trauma sensitive or trauma-sensitive or trauma services or complex trauma or trauma-base* or trauma base*).ab,ti.	2648
2	(trauma-inform* and (approach* or care or practi? or intervention* or system*)).ab,ti.	754
3	(trauma-inform* and (measure or survey or questionnaire or checklist or assessment or evaluation)).ab,ti.	279
4	or/1-3	2648
5	(adverse childhood event$ or adverse childhood experience$ or psychological* informed environment$).ab,ti.	1595
6	4 or 5	4152
7	(child or child* or adolescent*).mp.	2378418
8	child/ or infant/ or adolescent/	3091467
9	or/7-8	3568141
10	6 not 9	1468
