# Supplementary material for: Trauma-informed approaches to primary and community mental health care: protocol for a mixed-methods systematic review
Source: BMJ Open. 2021 Feb 18;11(2):e042112. doi: 10.1136/bmjopen-2020-042112 (PMC7896604; doi:10.1136/bmjopen-2020-042112)
Supplement: Author's manuscript [file bmjopen-2020-042112.draft_revisions.pdf]

BMJ Open

BMJ Open is committed to open peer review. As part of this commitment we make the peer review history of every article we publish publicly available.

When an article is published we post the peer reviewers' comments and the authors' responses online. We also post the versions of the paper that were used during peer review. These are the versions that the peer review comments apply to.

The versions of the paper that follow are the versions that were submitted during the peer review process. They are not the versions of record or the final published versions. They should not be cited or distributed as the published version of this manuscript.

BMJ Open is an open access journal and the full, final, typeset and author-corrected version of record of the manuscript is available on our site with no access controls, subscription charges or pay-per-view fees (http://bmjopen.bmj.com).

If you have any questions on BMJ Open's open peer review process please email info.bmjopen@bmj.com

BMJ Open

Trauma-informed approaches to primary and community mental health care: protocol for a systematic review

Journal:	*BMJ Open*
Manuscript ID	bmjopen-2020-042112
Article Type:	Protocol
Date Submitted by the Author:	25-Jun-2020

Complete List of Authors:	Dawson, Shoba; Centre for Academic Primary Care, Population Health Sciences, University of Bristol<br>Bierce, Angel; Centre for Academic Primary Care, Population Health Sciences, University of Bristol<br>Feder, Gene; Centre for Academic Primary Care, Population Health Sciences, University of Bristol<br>Macleod, John; Centre for Academic Primary Care, Population Health Sciences, University of Bristol and National Institute for Health Research Applied Research Collaboration West (ARC West), National Institute for Health Research (NIHR) Bristol Biomedical Research Centre, University Hospitals Bristol NHS Foundation Trust<br>Turner, Katrina; Centre for Academic Primary Care, Population Health Sciences, University of Bristol, National Institute for Health Research Applied Research Collaboration West (ARC West), NIHR Bristol Biomedical Research Centre, University Hospitals Bristol NHS Foundation Trust<br>Zammit, Stan; NIHR Bristol Biomedical Research Centre, University Hospitals Bristol NHS Foundation Trust and University of Bristol; Centre for Academic Mental Health, Population Health Sciences, ; Cardiff University, Division of Psychological Medicine and Clinical Neurosciences<br>Lewis, Natalia; Centre for Academic Primary Care, Population Health Sciences, University of Bristol and NIHR Bristol Biomedical Research Centre, University Hospitals Bristol NHS Foundation Trust

Keywords:	PRIMARY CARE, MENTAL HEALTH, PUBLIC HEALTH, TRAUMA MANAGEMENT

SCHOLARONE™
Manuscripts

BMJ

I, the Submitting Author has the right to grant and does grant on behalf of all authors of the Work (as defined in the below author licence), an exclusive licence and/or a non-exclusive licence for contributions from authors who are: i) UK Crown employees; ii) where BMJ has agreed a CC-BY licence shall apply, and/or iii) in accordance with the terms applicable for US Federal Government officers or employees acting as part of their official duties; on a worldwide, perpetual, irrevocable, royalty-free basis to BMJ Publishing Group Ltd ("BMJ") its licensees and where the relevant Journal is co-owned by BMJ to the co-owners of the Journal, to publish the Work in this journal and any other BMJ products and to exploit all rights, as set out in our licence.

The Submitting Author accepts and understands that any supply made under these terms is made by BMJ to the Submitting Author unless you are acting as an employee on behalf of your employer or a postgraduate student of an affiliated institution which is paying any applicable article publishing charge ("APC") for Open Access articles. Where the Submitting Author wishes to make the Work available on an Open Access basis (and intends to pay the relevant APC), the terms of reuse of such Open Access shall be governed by a Creative Commons licence – details of these licences and which Creative Commons licence will apply to this Work are set out in our licence referred to above.

Other than as permitted in any relevant BMJ Author's Self Archiving Policies, I confirm this Work has not been accepted for publication elsewhere, is not being considered for publication elsewhere and does not duplicate material already published. I confirm all authors consent to publication of this Work and authorise the granting of this licence.

For peer review only

Title Page

Trauma-informed approaches to primary and community mental health care: protocol for a systematic review

Dr Shoba Dawson. Centre for Academic Primary Care, Population Health Sciences, University of Bristol shoba.dawson@bristol.ac.uk

Mx Angel Bierce. Centre for Academic Primary Care, Population Health Sciences, University of Bristol fd19315@bristol.ac.uk

Professor Gene S Feder. Centre for Academic Primary Care, Population Health Sciences, University of Bristol Gene.Feder@bristol.ac.uk

Professor John Macleod. Centre for Academic Primary Care, Population Health Sciences, University of Bristol and National Institute for Health Research Applied Research Collaboration West (ARC West), National Institute for Health Research (NIHR) Bristol Biomedical Research Centre, University Hospitals Bristol NHS Foundation Trust John.MacLeod@bristol.ac.uk

Dr Katrina M Turner. Centre for Academic Primary Care, Population Health Sciences, University of Bristol, National Institute for Health Research Applied Research Collaboration West (ARC West), NIHR Bristol Biomedical Research Centre, University Hospitals Bristol NHS Foundation Trust Katrina.Turner@bristol.ac.uk

Professor Stanley Zammit. NIHR Bristol Biomedical Research Centre, University Hospitals Bristol NHS Foundation Trust and University of Bristol; Centre for Academic Mental Health, Population Health Sciences, University of Bristol; Division

For peer review only - http://bmjopen.bmj.com/site/about/guidelines.xhtml

of Psychological Medicine and Clinical Neurosciences, Cardiff University

Stan.Zammit@bristol.ac.uk

Dr Natalia V Lewis. Centre for Academic Primary Care, Population Health Sciences, University of Bristol and NIHR Bristol Biomedical Research Centre, University Hospitals Bristol NHS Foundation Trust nat.lewis@bristol.ac.uk

Correspondence to Dr Shoba Dawson, Centre for Academic Primary Care, Bristol Medical School, University of Bristol, 1.01 Canynge Hall, 39 Whatley Road, Bristol, BS8 2PS.

Keywords Trauma-informed approaches, systematic review, primary care, community mental health care

Wordcount 3088 (excluding title, abstract, table, figure and references)

For peer review only - http://bmjopen.bmj.com/site/about/guidelines.xhtml

Trauma-informed approaches to primary and community mental health care: protocol for a systematic review

Abstract

Introduction Exposure to different types of psychological trauma leads to a range of adverse effects on trauma survivors, including poorer mental and physical health, economic, social outcomes, and cognitive functioning. Trauma-informed (TI) approaches to care are defined as a service system grounded in and directed by an understanding of how trauma affects the survivors' neurological, biological, physiological, and social development. TI service system involves training of all staff, service improvements and sometimes screening for trauma experiences. The UK started incorporating TI approaches into the NHS. While policies recommend it, the evidence base for TI approaches to health care is not well established. We aim to conduct a systematic review to synthesise evidence on TI approaches in primary and community mental health care globally.

Methods and analysis We will undertake a systematic search for primary studies in Medline, Embase, PsycINFO, Cumulative Index to Nursing and Allied Health Literature, Cochrane library, websites of organisations involved in the development and implementation of TI approaches in health care, databases of thesis and dissertation. Included studies will be in English published between 2001 and February 2020. Two reviewers will independently perform study selection with data extraction and quality appraisal undertaken by one reviewer and checked for accuracy by second reviewer. A results-based convergent synthesis will be conducted where quantitative (narratively) and qualitative (thematically) evidence will be analysed separately and then integrated using another method of synthesis. We

For peer review only - http://bmjopen.bmj.com/site/about/guidelines.xhtml

For peer review only

set up a trauma survivor group and a professional group who were consulted throughout all stages of this review.

Ethics and dissemination There will be no requirement for ethical approval for this systematic review as no empirical data will be collected. The findings will be disseminated through a peer-reviewed publication, scientific and practitioner conferences, and policy briefings targeted at local and national policy makers.

PROSPERO registration number CRD42020164752

Article Summary

Strengths and limitations of this study

- This is the first systematic review of trauma-informed approaches in primary and community mental health care.
- The review will include peer reviewed and grey literature, providing a global view of trauma-informed approaches for informing health care services and future research in the UK.
- The involvement of people with lived experiences of trauma and healthcare professionals helps to produce evidence that is relevant to providers and recipients of health care and is therefore more likely to be translated into practice.
- One limitation of the review is that it will only include studies in the English language.
- The review will not include policy documents. This might be an area for future research.

For peer review only

For peer review only - http://bmjopen.bmj.com/site/about/guidelines.xhtml

Introduction

The Substance Abuse and Mental Health Services Administration (SAMHSA), which is the leading institution in the field of trauma informed (TI) care, defines trauma as "**event**, series of events, or set of circumstances that is **experienced** by an individual as physically or emotionally **harmful** or life-threatening and that have lasting adverse effects on the individual's functioning and mental, physical, social, emotional or spiritual well-being".[1] In the World Health Organisation (WHO) World Mental Health Survey, 70% of respondents experienced lifetime traumas, with exposure averaging 3.2 traumatic events per person. The most frequently reported traumas were those that occurred to a loved ones/witnessed (36%), those involving accidents (34%), unexpected death of loved ones (31%), physical violence (23%), intimate partner sexual violence (14%) and war-related traumas (13%).[2] In the English household survey, 47% of adults reported at least one adverse childhood experience (ACE). Prevalence of childhood sexual, physical, and verbal abuse was 6%, 15%, and 18% respectively.[3] According to the Crime Survey for England and Wales, 8% of women and 4% of men experienced domestic and sexual violence and abuse (DSVA) in the last year; lifetime prevalence is 29% and 13%, respectively.[4]

For peer review only

Experiencing traumas can have a wide range of adverse impacts on the victims, including poorer mental health, physical health, economic and social outcomes throughout the lifespan.[5] This means that a large proportion of people with health problems who access primary health care have experienced trauma in their lifetime. Primary health care is the first point of contact with health system for an individual. It plays a vital role in making health care universally accessible.[6] The WHO has adopted primary care as the preferred method for providing comprehensive,

For peer review only - http://bmjopen.bmj.com/site/about/guidelines.xhtml

equitable, affordable, and universal healthcare services for individuals and communities.[6] The WHO has made substantial investment to ensure that mental health services are integrated into primary care in the last decade.[7] The rationale for integration mental health services into primary care includes: reduced stigma, improved access to care, reduced chronicity and improved social integration, better health outcomes for people treated in primary care and improving human resource capacity for mental health.[7,8]

Several studies found strong evidence on the association between lifetime traumas and increased utilization of primary health care. The household survey in England and Wales found that adults with 4 ACEs were twice as likely to visit general practitioner 6 times or more in the last 12 months (OR 2.3, 95% CI 1.8-2.9) compared to adults with no ACEs.[9] The Australian Longitudinal Study on Women's Health found that women with lifetime violence experiences had almost twice the odds of higher general practice service use (AOR 1.82, 95% CI 1.37–2.40), compared with women without any violence experiences.[10] Majority of patients in community mental health and substance abuse services experienced repeated traumas throughout their lifespan.[11] Therefore, primary health care services must be designed in a way that will support the recovery of survivors. However, they often have the opposite effect and can trigger memories about traumatic experiences through invasive procedures and coercive practices (e.g., the removal of choice regarding treatment or judgemental attitudes following a disclosure of abuse; lack of available and acceptable services). Re-activation of traumatic experiences within health services can affect both service users and staff, with the latter experiencing vicarious trauma.[12]

For peer review only - http://bmjopen.bmj.com/site/about/guidelines.xhtml

The field of trauma-informed (TI) approaches (synonyms *TI care*, *TI practice, TI model of care, TI service system*) is relatively new. The framework of TI approaches was developed in the US,[13] where it is widely used across all sectors.[1] TI approaches can be defined as “a system of development model that is grounded and directed by a complete understanding of how trauma exposure affects service users’ neurological, biological, psychological and social development”. TI approaches to care shift the health care professional’s focus from diagnosis towards understanding trauma that lies at the root of symptoms and health conditions through:

1. Recognition. All people in an organisation recognise how patients’ and staff’s experiences of trauma might affect the way they think, feel and behave.
2. Realisation. All staff in the organisation accept how trauma can affect people, and patients behaviour is understood in the context of coping with their experiences.
3. Response. The organisation acts to effectively integrate knowledge about prevalence and impact of trauma into policies, procedures, and practices.
4. Resist re-activation of traumatic memories. Steps are taken to prevent further traumatising both service users and staff through focus on recovery of survivors, as well as the wellbeing of staff.[1]

A TI approach is distinct from trauma-specific interventions (e.g., trauma-focused cognitive behavioural therapy) or trauma services (e.g., Traumatic Stress Service) that treat trauma symptoms. A TI approach can include trauma-specific interventions, although the essential component is the incorporation of the above principles in the organisational/system levels.[1]

For peer review only - http://bmjopen.bmj.com/site/about/guidelines.xhtml

Most extant evidence for TI approaches comes from Northern America.[14-16] TI care has only recently been included in the UK National Health Service (NHS) long-term plan[17] and the NHS mental health implementation plan.[18] The Scottish Government[19] and the Safeguarding Board for Northern Ireland[20] endorsed TI approaches across health care, social care, education, and justice sectors. Public Health Wales published reports recommending TI approaches across public services.[21] TI approaches were endorsed in local governments policies across England.[22] However, the evidence base for TI approaches to health care is not well established. A recent scoping review identified only a few examples of TI care implemented in Scotland[19] and England[23,24] and recommended developing the evidence base to demonstrate the value of TI approaches in the UK healthcare context.[25]

The aim of this systematic review is to synthesize evidence on TI approaches in primary care and community mental health care globally, which will help inform the development of a UK specific model of TI in these settings. The synthesis will address the following research questions:

1. What models of TI care have been used in primary care and community mental health services?
2. What are the theories of change underpinning these models?
3. What evidence is available for the acceptability, effectiveness, and cost-effectiveness of TI approaches to primary care and community mental health care?

For peer review only - http://bmjopen.bmj.com/site/about/guidelines.xhtml

Methods and analysis

This protocol follows the Preferred Reporting Items for Systematic Review and Meta-Analysis Protocol (PRISMA-P) checklist (Appendix 1).[26] The systematic review will be conducted and reported following the PRISMA guidelines.[27]

Eligibility criteria

We will select studies according to the following criteria.

Population

Patients in primary and community mental health care (globally) aged 18 and over. Professionals who plan, commission, and deliver primary or community mental health care.

Intervention

We will use the SAMHSA definitions of TI approach[1]and include evaluations of any model of TI care in primary or community mental health organisations. The SAMHSA framework of TI care covers ten domains of organisational change in (1) governance and leadership, (2) written policies and protocols, (3) physical environment, (4) training and workforce development, (5) engagement and involvement of service users, (6) cross sector collaboration, (7) progress monitoring and quality assurance, (8) financing, (9) evaluation, (10) screening, assessment, and treatment for trauma. We will exclude studies of trauma-specific interventions that treat trauma symptoms.

Comparator

Primary and community mental health care not using TI approaches.

Outcomes

We identified outcomes from the prior literature on TI approaches to health care[14, 28-31] and consultations with two study advisory groups of trauma survivors and

For peer review only - http://bmjopen.bmj.com/site/about/guidelines.xhtml

professionals who plan, commission and deliver primary and community mental health care. To map the outcomes from all these sources on potential logic model of a TI care model, we adapted published measurement model for TI primary care (Figure 1).[31]

For peer review only

For peer review only - http://bmjopen.bmj.com/site/about/guidelines.xhtml

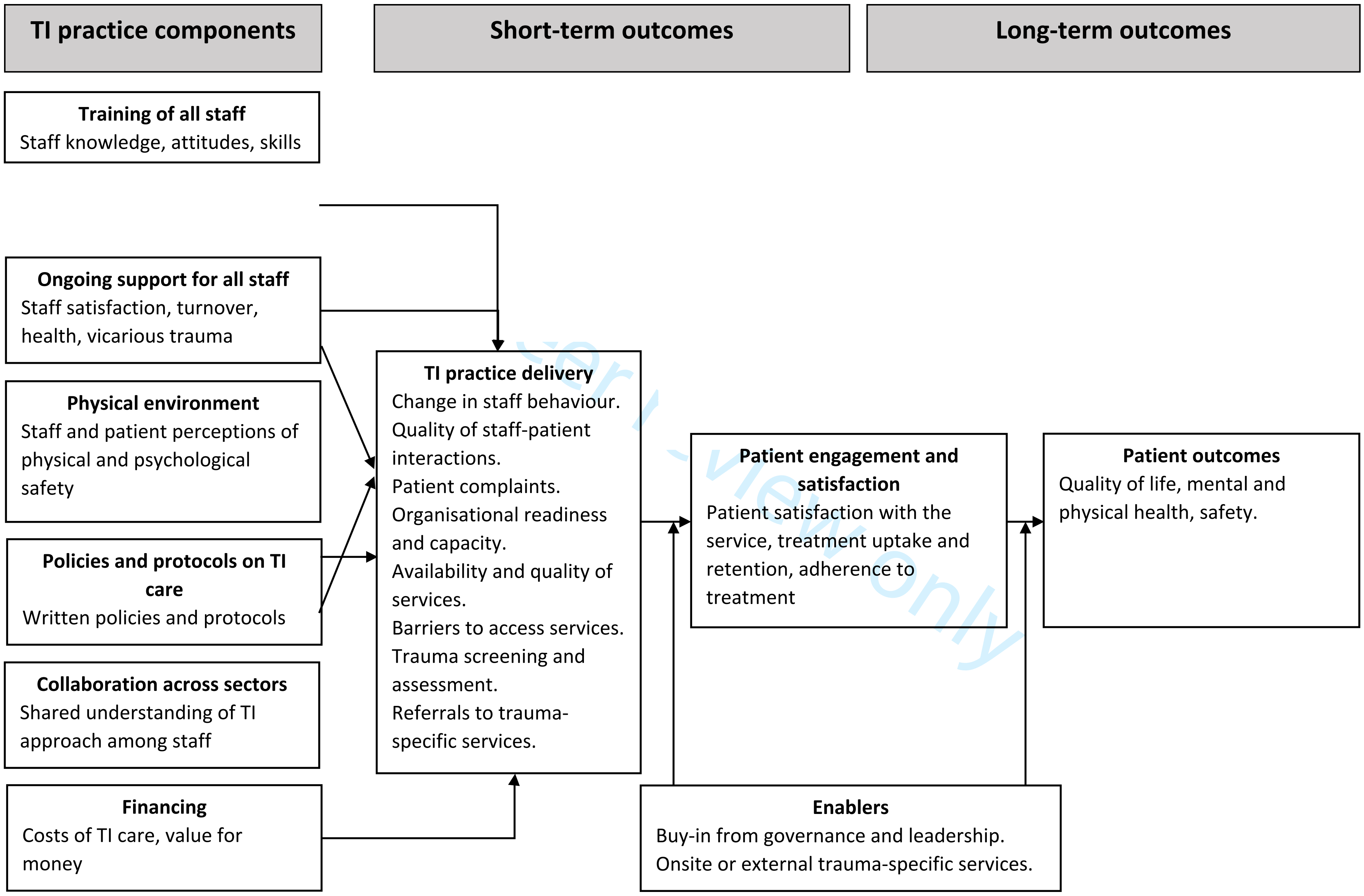

Figure 1: Outcomes for systematic review on trauma-informed primary and community mental health care. Adapted from German et al. (2020)[31]

For peer review only - http://bmjopen.bmj.com/site/about/guidelines.xhtml

To be included, a study must report a measure from at least one of the above outcome categories. The TI approach is an organization level intervention. Therefore, we define outcomes at organization level as main outcomes and outcomes at individual level as additional outcomes. We will also look at theories underpinning TI approaches. We will pay special attention to adverse effects of TI approaches on patient health, health care providers (e.g., vicarious trauma), service utilisation and quality of care.

Types of studies

Primary studies of any design that evaluated the acceptability and/or effectiveness and/or cost-effectiveness of TI approaches in primary and/or community mental health care will be included. Reference lists of systematic reviews that meet this criterion will be searched to identify relevant primary studies. Editorials, policy documents, and books will be excluded.

Setting

Any setting providing primary care including primary care mental health services. WHO defines primary health care centre as setting providing services that are usually the first point of contact with a health care professional.[32] Depending on country, they can include any open access, community based first point of care service, for example, general practice clinics, community-based units, basic health units, family health strategy, primary care home visits, day-care centres, multicentre health clinics, one stop crisis centre, Improving Access to Psychological Therapies (IAPT) services.[8]

For peer review only

For peer review only - http://bmjopen.bmj.com/site/about/guidelines.xhtml

Time frame

An early and influential paper[13] discussing TI approaches was published in 2001. We will limit the studies by date to ensure that the search identifies all relevant studies since this publication.

Language

There will be no language restrictions, provided an English language abstract is available for initial screening.

Search strategy

SD and NVL will develop a comprehensive search strategy using a combination of MeSH and free-text terms, based on previous systematic reviews in the areas of TI care[29,33] and the expertise of the research team to identify relevant papers on TI approaches in primary and community mental health care for adults. SD will run several scoping exercises in different electronic databases to maximise the sensitivity and specificity of the developed search strategy (Appendix 2, example search strategy).

SD will search electronic bibliographic databases for potential primary studies from January 2001- February 2020: Cochrane Library, MEDLINE, EMBASE, Cumulative Index to Nursing and Allied Health Literature (CINAHL EBSCO) and PsycINFO and update searches in the last two months of the study.

In addition, SD will search the PROSPERO database to identify any relevant systematic reviews in progress. She will also conduct a grey literature search to identify studies not indexed in the databases listed above. SD will search websites of organisations involved in development and implementation of TI care: UK national and local governments, King's Fund, SAMHSA, Violence, Abuse and Mental Health

For peer review only - http://bmjopen.bmj.com/site/about/guidelines.xhtml

Network and Trello (Adverse Childhood Experiences Resource library). Theses and dissertations will be identified through ethos library and PROQUEST. SD, AB, NVL will carry out forward and backward referencing of included papers to supplement the database and grey literature searches to identify any further relevant articles. SD and NVL will approach corresponding authors of included papers, study advisory groups, and experts in the field of trauma and primary care for additional relevant articles.

Screening of studies

References will be managed in Rayyan (https://rayyan.qcri.org/). SD will export search results from the different databases into the Rayyan database and remove duplicates. Study selection will be completed in two stages: first, titles and abstracts will be screened; next full-text will be screened to identify studies eligible for inclusion. Two members of the research team (SD and AB or NVL) will independently screen titles, abstracts and full-text. Any discrepancies between reviewers will be discussed with other team members.

We will list excluded full text studies in the table categorised by reasons for exclusion. We will collate multiple reports of the same study so that each study, rather than each report, is the unit of analysis in the review. We will also provide any information we can obtain from corresponding authors about ongoing studies. We will record the screening process in detail to complete a PRISMA flow diagram.[34]

Data extraction

A data extraction form will be used to focus on the characteristics that are relevant to this review:

For peer review only - http://bmjopen.bmj.com/site/about/guidelines.xhtml

For peer review only

1. Methods: type of study (randomised trial, interrupted time series, controlled/uncontrolled before-after, cross-sectional, qualitative, mixed-method, service evaluation).
2. Study setting (country, key features of the health care system, health care setting)
3. Characteristics of the participants
4. Characteristics of the TI approach: components, comparison. We will map components of each TI model on the SAMHSA's ten-domains framework of organisational change[1]
5. Outcomes: main and additional outcomes specified and collected, time points reported
6. Theories underpinning TI approach

We will seek input from the study advisory groups of trauma survivors and professionals on any other relevant data that should be extracted. SD will pilot the adapted extraction form on publications of a quantitative, qualitative and mixed-method study, and then refine it. To minimise bias and errors, one reviewer (SD) will extract the data and a second reviewer (AB or NVL) will check the extraction in detail. Any disagreements between reviewers will be resolved through discussions and, if required, with other team members.

Quality appraisal

We will use the Critical Appraisal Skill Programme (CASP) tools for different study designs.[35] Quality appraisal will be carried out as part of data extraction. SD will complete CASP checklists for each study. Second reviewer (AB or NVL) will check completed checklists in detail. Any disagreement between the reviewers will be resolved through discussions and if required by other members of the team.

For peer review only - http://bmjopen.bmj.com/site/about/guidelines.xhtml

Synthesis

Based on feasibility searches and background reading we expect that included quantitative studies will report outcomes that vary substantially by the way they were defined and measured. For this reason, we anticipate that a quantitative synthesis of the results from the quantitative studies will not be appropriate. We will, therefore, employ narrative synthesis to summarise findings from quantitative studies. This will involve the use of descriptive text and tables to summarise data to allow readers to consider findings in the light of differences in study designs. We will describe all TI models in a table based on the TiDIER template.[36] For each model of TI care, we will describe the range of effects found in the studies and if possible, the theory of change through which the TI models were intended to affect specific outcomes. For qualitative studies, we plan to use the thematic synthesis method.[37] Findings from the quantitative and qualitative synthesis will be integrated using a results-based convergent synthesis design. We will analyse quantitative and qualitative evidence separately and then integrate it using another method of synthesis. This approach allows for comparison of quantitative and qualitative evidence using tables and matrices considering the results from both the synthesis.[38]

Discussion

An effective response to (often) hidden trauma of patients in general practice and primary care mental health services is long overdue. This is a protocol for a systematic review on TI approaches in primary care and community mental health care that addresses gap in evidence on the acceptability, effectiveness, and cost-effectiveness of TI health care. The study includes both peer-reviewed and grey literature and offers a global view of TI approaches. Our findings will inform

For peer review only

development of an evidence-based UK-specific model of TI primary care and community mental health care. Although the output of this review will form basis for further research, the findings will be relevant to current policy and practice, even before we have developed and tested the UK-specific TI model. UK policy makers can use this new evidence when developing/amending health policies on TI care. Involvement of two advisory groups of trauma survivors and providers of health care throughout all stages of this review helps to produce evidence that is relevant to end users and is likely to be translated into policy and practice.

A limitation of this study is that it will only include studies in the English language. Policy documents will be excluded from this review and is an area for future policy review. This study was conceived and designed at pre-COVID-19 pandemic era and will be delivered and disseminated throughout and after the pandemic. The WHO[39] and statutory and third sector organisations[40,41] have already reported that stress, social isolation measures, quarantines at home, etc. resulted in increase of all forms of family violence. Consultation with our advisory groups on the impact of the pandemic has supported these findings. Our lay and professional contributors talked about rise in traumatic experiences among patients and health care professionals, worsening mental health, increased demand for health and social services and transition of services from face-to-face delivery to phone or online delivery mode. This changing environment makes our systematic review of TI approaches to health care timely in providing evidence for effective and acceptable primary and community mental health care in the post-pandemic era.

For peer review only - http://bmjopen.bmj.com/site/about/guidelines.xhtml

Patient and Public Involvement

We have set up two advisory groups to work with researchers throughout the study: a trauma survivor group and a professional group. The trauma survivor advisory group consists of eight people with diverse lived experiences of trauma who had been recipients of care in the NHS and other care systems. The professional advisory group consists of eight professionals (e.g. planning and development manager from local authority, clinical psychologist, clinical lead specialist) from England and Wales who are involved in planning, funding, commissioning, or delivering primary health care or community mental health services.

We organised separate meetings with survivor and professional advisers where they were introduced to the study and systematic review process. Both groups took part in brainstorming exercises on formulating research questions and listing outcomes for the systematic review that are meaningful to patients, practitioners, service managers and commissioners. Based on their feedback, SD and NL mapped the outcomes to those identified in the existing literature independently and any discrepancies were resolved through discussions. The professional advisory group developed a list of UK primary and community mental health services. The group highlighted inconsistent terminology used in the UK (i.e., ACEs, TI approaches/care/practice, psychologically informed environments) and diverse approaches to developing policies on TI care across the UK (i.e., top down in Scotland, Northern Ireland, and Wales vs bottom up in England). They highlighted the need for synthesised research evidence about such approaches that is relevant to the UK and for using this evidence to inform health policy. We will meet with the survivor and professional advisory groups biannually to consult on data extraction, interpretation, and dissemination of study findings.

For peer review only - http://bmjopen.bmj.com/site/about/guidelines.xhtml

Ethics and dissemination

No ethical approval is required for this systematic review as this does not involve collection of primary data. Findings of this review will be disseminated through publication of a peer-reviewed paper, papers presented at conferences for academic and practitioner audiences, through local clinical commissioning groups and policy briefings targeted at local and national policy makers. Our survivor and professional advisers will be consulted for sources through which we should disseminate the findings. We will also work with survivor advisers to develop a lay summary of the study findings which will be disseminated to Centre for Academic Primary Care PPI contributors.

Acknowledgements

We would like to thank our survivor and professional advisory group for their contribution in identifying outcomes for this systematic review.

Author contributions

NVL conceived the idea and secured funding. NVL and SD designed the study. SD drafted study protocol. SD, AB and NVL set up and coordinate study advisory groups with support from Mike Bell (BRC PPI coordinator). SD and NVL produced first draft of the manuscript. All co-authors edited it and approved the version for submission.

Funding statement

This study is funded by the NIHR Biomedical Research Centre at University Hospitals Bristol NHS Foundation Trust and the University of Bristol. Grant reference number R100514-105. The views expressed are those of the authors and not necessarily those of the NIHR or the Department of Health and Social Care.

For peer review only - http://bmjopen.bmj.com/site/about/guidelines.xhtml

Competing interests statement

Authors have no conflict of interest to declare.

For peer review only

References

1. Substance Abuse and Mental Health Services Administration (SAMHSA). *SAMHSA's concept of trauma and guidance for a trauma-informed approach*. SAMHSA; 2017. https://store.samhsa.gov/system/files/sma14-4884.pdf. p. 27. Accessed 10th February 2020.
2. Kessler RC et al. Trauma and PTSD in the WHO World Mental Health Surveys. *Eur J Psychotraumatol*, 2017; **8**:5; p. 1353383.
3. Bellis MA et al. National household survey of adverse childhood experiences and their relationship with resilience to health-harming behaviors in England. *BMC Med.* 2014; **12**: p. 72.
4. Office for National Statistics. *Domestic abuse in England and Wales: year ending March 2018*; 2018. https://www.ons.gov.uk/peoplepopulationandcommunity/crimeandjustice/bulletins/domesticabuseinenglandandwales/yearendingmarch2018#prevalence-of-domestic-abuse. Accessed 10th February 2020.
5. Magruder KM, McLaughlin KA et al. Trauma is a public health issue. *Eur J Psychotraumatol*, 2017; **8**(1): p. 1375338.
6. World Health Organization. Declaration of alma-ata: World Health Organization, Regional Office for Europe: WHO, 2004.
7. World Health Organisation. Integrating mental health services into primary health care. Geneva: WHO, 2007 (http://www.who.int/mental_health/policy/services/en/index.html ; Mental Health Policy, Planning and Service Development Information Sheet, Sheet3). Accessed 9th May 2020.

For peer review only - http://bmjopen.bmj.com/site/about/guidelines.xhtml

8. Charles A, Ham C et al. Reimagining community services. Making the most of our assets. The King's Fund, 2018: Accessed 4th May 2020. https://www.kingsfund.org.uk/publications/community-services-assets. p. 118.
9. Bellis M, Hughes K et al. The impact of adverse childhood experiences on health service use across the life course using a retrospective cohort study. *J of Health Serv Res & Pol*. 2017; **22**(3): p. 168-177.
10. Coles J, Lee A. et al. General practice service use and satisfaction among female survivors of childhood sexual abuse. *Aust Fam Physician*. 2015; **44**(1): p. 71-76.
11. Rosenberg L. (2011) Addressing Trauma in Mental Health and Substance Use Treatment. *Journal of Beh Health Serv & Res*. 2011; **38**(4): p. 428-431.
12. Figley CR, Kleber RJ. *Beyond the "Victim"*. In: Kleber R.J., Figley C.R., Gersons B.P.R. (eds) Beyond Trauma. The Plenum Series on Stress and Coping. 1995; Springer, Boston, MA.
13. Harris M and Fallot R, *Using trauma theory to design service systems*. 2001, San Francisco, CA: Jossey-Bass.
14. Melz H, Morrison C et al., (2019) Review of trauma-informed initiatives at the systems level. Trauma-informed approaches: connecting research, policy, and practice to build resilience in children and families. 2019. https://aspe.hhs.gov/system/files/pdf/262051/TI_Approaches_Research_Review.pdf. p.37; Accessed 18th January 2020.
15. Raja S, Hasnain M et al. Trauma informed care in medicine: current knowledge and future research directions. *Fam Community Health*. 2015; **38**(3): p. 216-226.
16. Reeves E. A synthesis of the literature on trauma-informed care. *Issues Ment Health Nurs*. 2015. **36**(9): p. 698-709.

For peer review only - http://bmjopen.bmj.com/site/about/guidelines.xhtml

17. NHS. The NHS Long Term Plan. 2019. https://www.longtermplan.nhs.uk/wp-content/uploads/2019/08/nhs-long-term-plan-version-1.2.pdf, Accessed 20th January 2020.
18. NHS. Mental Health Implementation Plan 2019/20 – 2023/24. 2019. https://www.longtermplan.nhs.uk/wp-content/uploads/2019/07/nhs-mental-health-implementation-plan-2019-20-2023-24.pdf, Accessed 20th January 2020.
19. NHS Education for Scotland. Transforming psychological trauma: a knowledge and skills framework for the Scottish workforce. 2017. https://www.nes.scot.nhs.uk/media/3971582/nationaltraumatrainingframework.pdf Accessed 20th January 2020.
20. Bunting L, Montgomery L et al., Developing trauma informed practice in Northern Ireland: Key messages. 2019, Queen's University, Belfast: https://pureadmin.qub.ac.uk/ws/portalfiles/portal/168356931/ACEs_Report_A4_Feb_2019_Key_Messages.pdf p. 33. Accessed 20th January 2020.
21. Public Health Wales NHS Trust. Adverse Childhood Experiences and their association with chronic disease and health service use in the Welsh adult population. Walsh Adverse Childhood Experiences (ACE) study. 2016. http://www.wales.nhs.uk/sitesplus/documents/888/ACE%20Chronic%20Disease%20report%20%289%29%20%282%29.pdf. Accessed 25th May 2020.
22. Greater London Authority. Connecting up the care. Supporting London's children exposed to domestic abuse, parental mental ill-health and parental substance abuse. 2020. https://www.london.gov.uk/sites/default/files/connecting_up_the_care.pdf. Accessed 25th May 2020

For peer review only

23. Trauma-informed care. 2019. https://www.tewv.nhs.uk/services/trauma-informed-care/. Accessed 28th January 2020.
24. Petrillo M et al. Healing Trauma evaluation report. 2019: https://researchportal.port.ac.uk/portal/files/14351866/HT_evaluation_full_report_June_2019.pdf. Accessed 28th January 2020.
25. Sweeney A, Clement S et al. Trauma-informed mental healthcare in the UK: what is it and how can we further its development? *Mental Health Review Journal*. 2016; **21**(3): p. 174-192.
26. Shamseer L, Moher D, Clarke M et al. Preferred reporting items for systematic review and meta-analysis protocols (PRISMA-P) 2015: elaboration and explanation. *BMJ* 2015; **350**: g7647.
27. Moher D, Liberati A, Tetzlaff J et al. Preferred reporting items for systematic reviews and meta-analyses: the PRISMA statement. *BMJ* 2009; **339**: b2535.
28. The Scottish Government. Survivor Scotland. Strategic outcomes and priorities 2015-17. 2015. https://www.gov.scot/publications/survivorscotland-strategic-outcomes-priorities-2015-2017/pages/1/, Accessed 21st February 2020.
29. Champine RB, Lang JM et al. Systems Measures of a Trauma-Informed Approach: A Systematic Review. *Am J Community Psychol.* 2019; **64**(3): p. 418-437.
30. Robotham D, Sweeney A et al. Violence, Abuse and Mental Health Network. Survivors' priority themes and questions for research. *Consultation Report*. 2019, Violence, Abuse and Mental Health Network: https://www.vamhn.co.uk/uploads/1/2/2/7/122741688/consultation_report_on_website.pdf, Accessed 21st February 2020.

For peer review only - http://bmjopen.bmj.com/site/about/guidelines.xhtml

31. Germán M et al. Measuring the impact of trauma-informed primary care: are we missing the forest for the trees? 2020, Montefiore Medical Group: https://www.chcs.org/resource/measuring-the-impact-of-trauma-informed-primary-care-are-we-missing-the-forest-for-the-trees/. p.15. Accessed 12th May 2020.
32. World Health Organisation. A vision for primary health care in the 21st century: towards universal health coverage and the Sustainable Development Goals: Geneva: WHO, 2018. https://www.who.int/docs/default-source/primary-health/vision.pdf. Accessed 12th May 2020.
33. Branson CE, Baetz CL et al. Trauma-Informed Juvenile Justice Systems: A Systematic Review of Definitions and Core Components. *Psychol Trauma:* 2017; **9**(6): p. 635-646.
34. Liberati A, Altman DG et al. The PRISMA statement for reporting systematic reviews and meta-analyses of studies that evaluate health care interventions: explanation and elaboration. *BMJ.* 2009; **339**: b2700.
35. Critical Appraisal Skills Programme (CASP). 2019. Critical Appraisal Skills Programme (CASP). https://casp-uk.net/casp-tools-checklists/, Accessed 16th January 2020.
36. Hoffmann TC, Glasziou PP et al. Better reporting of interventions: template for intervention description and replication (TIDieR) checklist and guide. *BMJ*. 2014; **348**: p. g1687.
37. Thomas J. and Harden A. Methods for the thematic synthesis of qualitative research in systematic reviews. *BMC Med Res Methodol*. 2008; **8**: p. 45.

For peer review only - http://bmjopen.bmj.com/site/about/guidelines.xhtml

38. Hong QN, Pluye P et al. Convergent and sequential synthesis designs: implications for conducting and reporting systematic reviews of qualitative and quantitative evidence. *Syst Rev. 2017;* **6** (1): 61.

39. World Health Organisation. COVID-19 and violence against women. What the health sector/system can do. WHO, 2020. https://www.who.int/reproductivehealth/publications/VAW-COVID19-Rev1.pdf. p. 3. Accessed 25th May 2020.

40. Women's Aid. The impact of COVID-19 on women and children experiencing domestic abuse, and the life-saving services that support them. 2020. https://www.womensaid.org.uk/the-impact-of-covid-19-on-women-and-children-experiencing-domestic-abuse-and-the-life-saving-services-that-support-them/, Accessed 13th April 2020.

41. UNICEF. The Alliance for Child Protection in Humanitarian Action, Technical Note: Protection of Children during the Coronavirus Pandemic. 2020. https://www.unicef.org/documents/technical-note-protection-children-coronavirus-disease-2019-covid-19-pandemic, Accessed 30th May 2020.

For peer review only

For peer review only - http://bmjopen.bmj.com/site/about/guidelines.xhtml

Appendix 1: Preferred Reporting Items for Systematic review and Meta-Analysis Protocols (PRISMA-P) checklist (Shamseer et al. 2015)[26]

Section and topic	Item No	Checklist item	Reported on Page #
**Administrative Information**			
**Title**			
Identification	1a	Identify the report as a protocol of a systematic review	1 (Title)
Update	1b	If the protocol is for an update of a previous systematic review, identify as such	NA
Registration	2	If registered, provide the name of the registry (e.g., PROSPERO) and registration number in the Abstract	4 (Abstract)
**Authors**			
Contact	3a	Provide name, institutional affiliation, and e-mail address of all protocol authors; provide physical mailing address of corresponding author	1-2
Contributions	3b	Describe contributions of protocol authors and identify the guarantor of the review	19 (Author Contributions)
Amendments	4	If the protocol represents an amendment of a previously completed or published protocol, identify as such and list changes; otherwise, state plan for documenting important protocol amendments	NA
**Support**			
Sources	5a	Indicate sources of financial or other support for the review	19 (Funding statement)
Sponsor	5b	Provide name for the review funder and/or sponsor	19 (Funding statement)
Role of sponsor/funder	5c	Describe roles of funder(s), sponsor(s), and/or institution(s), if any, in developing the protocol	NA
**Introduction**			
Rationale	6	Describe the rationale for the review in the context of what is already known	5-8 (Introduction)
Objectives	7	Provide an explicit statement of the question(s) the review will address with reference to participants, interventions, comparators, and outcomes (PICO)	8 (last paragraph), 9-13 (PICO)
**Methods**			
Eligibility criteria	8	Specify the study characteristics (e.g., PICO, study design, setting, time frame) and report characteristics (e.g., years considered, language, publication status) to be used as criteria for eligibility for the review	9-13 (eligibility criteria)

For peer review only - http://bmjopen.bmj.com/site/about/guidelines.xhtml

Information sources	9	Describe all intended information sources (e.g., electronic databases, contact with study authors, trial registers, or other grey literature sources) with planned dates of coverage	13-14 (search strategy, paragraph 2-3)
Search strategy	10	Present draft of search strategy to be used for at least one electronic database, including planned limits, such that it could be repeated	Appendix 2 (Supplementary file)
Study records			
Data management	11a	Describe the mechanism(s) that will be used to manage records and data throughout the review	14 (Screening of studies, paragraph 1)
Selection process	11b	State the process that will be used for selecting studies (e.g., two independent reviewers) through each phase of the review (i.e., screening, eligibility, and inclusion in meta-analysis)	14 (Screening of studies, paragraph 1)<br>15 (Data extraction, paragraph 2)
Data collection process	11c	Describe planned method of extracting data from reports (e.g., piloting forms, done independently, in duplicate), any processes for obtaining and confirming data from investigators	15 (Data extraction, paragraph 2)
Data items	12	List and define all variables for which data will be sought (e.g., PICO items, funding sources), any pre-planned data assumptions and simplifications	14 and 15 (Data extraction)
Outcomes and prioritization	13	List and define all outcomes for which data will be sought, including prioritization of main and additional outcomes, with rationale	11 (Figure 1)
Risk of bias in individual studies	14	Describe anticipated methods for assessing risk of bias of individual studies, including whether this will be done at the outcome or study level, or both; state how this information will be used in data synthesis	15 (Quality appraisal)
Data Synthesis	15a	Describe criteria under which study data will be quantitatively synthesized	NA
	15b	If data are appropriate for quantitative synthesis, describe planned summary measures, methods of handling data, and methods of combining data from studies, including any planned exploration of consistency (e.g., I^2, Kendall's tau)	NA
	15c	Describe any proposed additional analyses (e.g., sensitivity or subgroup analyses, meta-regression)	NA
	15d	If quantitative synthesis is not appropriate, describe the type of summary planned	16 (Synthesis)
Meta-bias(es)	16	Specify any planned assessment of meta-bias(es) (e.g., publication bias across studies, selective reporting within studies)	NA
Confidence in cumulative evidence	17	Describe how the strength of the body of evidence will be assessed (e.g., GRADE)	NA

*NA- Not Applicable

For peer review only - http://bmjopen.bmj.com/site/about/guidelines.xhtml

Appendix 2: Example search strategy

Database(s): **Ovid MEDLINE(R)** 1946 to present

Search Strategy:

#	Searches	Results
1	(trauma inform* or trauma-inform* or trauma focus* or trauma-focus* or trauma responsive* or trauma sensitive or trauma-sensitive or trauma services or complex trauma or trauma-base* or trauma base*).ab,ti.	2648
2	(trauma-inform* and (approach* or care or practi? or intervention* or system*)).ab,ti.	754
3	(trauma-inform* and (measure or survey or questionnaire or checklist or assessment or evaluation)).ab,ti.	279
4	or/1-3	2648
5	(adverse childhood event$ or adverse childhood experience$ or psychological* informed environment$).ab,ti.	1595
6	4 or 5	4152
7	(child or child* or adolescent*).mp.	2378418
8	child/ or infant/ or adolescent/	3091467
9	or/7-8	3568141
10	6 not 9	1468
11	limit 10 to yr="2001 -Current"	1347

only

BMJ Open

Trauma-informed approaches to primary and community mental health care: protocol for a mixed-methods systematic review

Journal:	*BMJ Open*
Manuscript ID	bmjopen-2020-042112.R1
Article Type:	Protocol
Date Submitted by the Author:	12-Jan-2021

Complete List of Authors:	Dawson, Shoba; Centre for Academic Primary Care, Population Health Sciences, University of Bristol<br>Bierce, Angel; Centre for Academic Primary Care, Population Health Sciences, University of Bristol<br>Feder, Gene; Centre for Academic Primary Care, Population Health Sciences, University of Bristol<br>Macleod, John; Centre for Academic Primary Care, Population Health Sciences, University of Bristol and National Institute for Health Research Applied Research Collaboration West (ARC West), National Institute for Health Research (NIHR) Bristol Biomedical Research Centre, University Hospitals Bristol NHS Foundation Trust<br>Turner, Katrina; Centre for Academic Primary Care, Population Health Sciences, University of Bristol, National Institute for Health Research Applied Research Collaboration West (ARC West), NIHR Bristol Biomedical Research Centre, University Hospitals Bristol NHS Foundation Trust<br>Zammit, Stan; NIHR Bristol Biomedical Research Centre, University Hospitals Bristol NHS Foundation Trust and University of Bristol; Centre for Academic Mental Health, Population Health Sciences, ; Cardiff University, Division of Psychological Medicine and Clinical Neurosciences<br>Lewis, Natalia; Centre for Academic Primary Care, Population Health Sciences, University of Bristol and NIHR Bristol Biomedical Research Centre, University Hospitals Bristol NHS Foundation Trust

**Primary Subject Heading**:	Health services research
Secondary Subject Heading:	Health services research
Keywords:	PRIMARY CARE, MENTAL HEALTH, PUBLIC HEALTH, TRAUMA MANAGEMENT

SCHOLARONE™ Manuscripts

I, the Submitting Author has the right to grant and does grant on behalf of all authors of the Work (as defined in the below author licence), an exclusive licence and/or a non-exclusive licence for contributions from authors who are: i) UK Crown employees; ii) where BMJ has agreed a CC-BY licence shall apply, and/or iii) in accordance with the terms applicable for US Federal Government officers or employees acting as part of their official duties; on a worldwide, perpetual, irrevocable, royalty-free basis to BMJ Publishing Group Ltd ("BMJ") its licensees and where the relevant Journal is co-owned by BMJ to the co-owners of the Journal, to publish the Work in this journal and any other BMJ products and to exploit all rights, as set out in our licence.

The Submitting Author accepts and understands that any supply made under these terms is made by BMJ to the Submitting Author unless you are acting as an employee on behalf of your employer or a postgraduate student of an affiliated institution which is paying any applicable article publishing charge ("APC") for Open Access articles. Where the Submitting Author wishes to make the Work available on an Open Access basis (and intends to pay the relevant APC), the terms of reuse of such Open Access shall be governed by a Creative Commons licence – details of these licences and which Creative Commons licence will apply to this Work are set out in our licence referred to above.

Other than as permitted in any relevant BMJ Author's Self Archiving Policies, I confirm this Work has not been accepted for publication elsewhere, is not being considered for publication elsewhere and does not duplicate material already published. I confirm all authors consent to publication of this Work and authorise the granting of this licence.

For peer review only

Title Page

Trauma-informed approaches to primary and community mental health care: protocol for a mixed-methods systematic review

Dr Shoba Dawson. Centre for Academic Primary Care, Population Health Sciences, University of Bristol shoba.dawson@bristol.ac.uk

Mx Angel Bierce. Centre for Academic Primary Care, Population Health Sciences, University of Bristol fd19315@bristol.ac.uk

Professor Gene S Feder. Centre for Academic Primary Care, Population Health Sciences, University of Bristol Gene.Feder@bristol.ac.uk

Professor John Macleod. Centre for Academic Primary Care, Population Health Sciences, University of Bristol and National Institute for Health Research Applied Research Collaboration West (ARC West), National Institute for Health Research (NIHR) Bristol Biomedical Research Centre, University Hospitals Bristol NHS Foundation Trust John.MacLeod@bristol.ac.uk

Dr Katrina M Turner. Centre for Academic Primary Care, Population Health Sciences, University of Bristol, National Institute for Health Research Applied Research Collaboration West (ARC West), NIHR Bristol Biomedical Research Centre, University Hospitals Bristol NHS Foundation Trust Katrina.Turner@bristol.ac.uk

Professor Stan Zammit. NIHR Bristol Biomedical Research Centre, University Hospitals Bristol NHS Foundation Trust and University of Bristol; Centre for Academic Mental Health, Population Health Sciences, University of Bristol; Division

For peer review only - http://bmjopen.bmj.com/site/about/guidelines.xhtml

of Psychological Medicine and Clinical Neurosciences, Cardiff University

Stan.Zammit@bristol.ac.uk

Dr Natalia V Lewis. Centre for Academic Primary Care, Population Health Sciences, University of Bristol and NIHR Bristol Biomedical Research Centre, University Hospitals Bristol NHS Foundation Trust nat.lewis@bristol.ac.uk

Correspondence to Dr Shoba Dawson, University of Bristol Medical School, Centre for Academic Primary Care, 1.01 Canynge Hall, 39 Whatley Road, Bristol, BS8 2PS.

Keywords Trauma-informed approaches, systematic review, primary care, community mental health care

Wordcount 3744 (excluding title, abstract, table, figure and references)

For peer review only

For peer review only - http://bmjopen.bmj.com/site/about/guidelines.xhtml

Trauma-informed approaches to primary and community mental health care: protocol for a mixed-methods systematic review

Abstract

Introduction Exposure to different types of psychological trauma may lead to a range of adverse effects on trauma survivors, including poor mental and physical health, economic, social, and cognitive functioning outcomes. Trauma-informed (TI) approaches to care are defined as a service system grounded in and directed by an understanding of how trauma affects the survivors' neurological, biological, physiological, and social development. TI service system involves training of all staff, service improvements and sometimes screening for trauma experiences. The UK started incorporating TI approaches into the National Health Service. While policies recommend it, the evidence base for TI approaches to health care is not well established. We aim to conduct a systematic review to synthesise evidence on TI approaches in primary and community mental health care globally.

Methods and analysis We will undertake a systematic search for primary studies in Medline, Embase, PsycINFO, Cumulative Index to Nursing and Allied Health Literature, Cochrane library, websites of organisations involved in the development and implementation of TI approaches in health care, and databases of thesis and dissertation. Included studies will be in English published between 1990 and February 2020. Two reviewers will independently perform study selection with data extraction and quality appraisal undertaken by one reviewer and checked for accuracy by a second reviewer. A results-based convergent synthesis will be conducted where quantitative (narratively) and qualitative (thematically) evidence will be analysed separately and then integrated using another method of synthesis. We

For peer review only - http://bmjopen.bmj.com/site/about/guidelines.xhtml

set up a trauma survivor group and a professional group to consult throughout this review.

Ethics and dissemination There is no requirement for ethical approval for this systematic review as no empirical data will be collected. The findings will be disseminated through a peer-reviewed publication, scientific and practitioner conferences, and policy briefings targeted at local and national policy makers.

PROSPERO registration number CRD42020164752

Article Summary

Strengths and limitations of this study

- This is the first mixed-methods systematic review of trauma-informed approaches in primary and community mental health care.
- The review will include peer-reviewed and grey literature, providing a global view of trauma-informed approaches for informing health care services and future research in the UK.
- The involvement of people with lived experiences of trauma and healthcare professionals helps to produce evidence that is relevant to providers and recipients of health care and is therefore, more likely to be translated into practice.
- One limitation of the review is that it will exclude studies without an abstract in English which could lead to missing relevant studies.
- The review will not include policy documents. This might be an area for future research.

For peer review only

Introduction

The Substance Abuse and Mental Health Services Administration (SAMHSA), which is the leading institution in the field of trauma-informed (TI) care, defines individual trauma as an “**event**, series of events, or set of circumstances that is **experienced** by an individual as physically or emotionally **harmful** or life-threatening and that have lasting adverse effects on the individual’s functioning and mental, physical, social, emotional or spiritual well-being”.[1] According to the World Health Organisation (WHO) World Mental Health Survey, 70% of respondents experienced lifetime traumas, with exposure averaging 3.2 traumatic events per person. The most frequently reported traumas were those that occurred to loved ones/witnessed (36%), those involving accidents (34%), unexpected death of loved ones (31%), physical violence (23%), intimate partner sexual violence (14%) and war-related traumas (13%).[2] In the English household survey, 47% of adults reported at least one adverse childhood experience (ACE). Prevalence of childhood sexual, physical, and verbal abuse was 6%, 15%, and 18%, respectively.[3] According to the Crime Survey for England and Wales, 8% of women and 4% of men experienced domestic and sexual violence and abuse (DSVA) in the last year; lifetime prevalence was 29% and 13%, respectively.[4]

Experiencing trauma can have a wide range of adverse impacts on the victims, including poorer mental health, physical health, economic and social outcomes throughout the life span.[5] This means that a large proportion of people with health problems who access primary health care and community mental health care have experienced trauma in their lifetime. Primary health care and community mental health care is the first point of contact with a health system for an individual. It plays

For peer review only - http://bmjopen.bmj.com/site/about/guidelines.xhtml

a vital role in making health care universally accessible.[6] The WHO has adopted primary care as the preferred method for providing comprehensive, equitable, affordable, and universal healthcare services for individuals and communities.[6] The WHO has made a substantial investment to ensure that mental health services are integrated into primary care in the last decade.[7] The rationale for the integration of mental health services into primary care includes: reduced stigma, improved access to care, reduced chronicity and improved social integration, better health outcomes for people treated in primary care and improved human resource capacity for mental health.[7,8] Both primary health care and community mental health care provide more accessible outpatient services. Both deal with patients who have co-occurring conditions and multiple health and social needs. Both integrate patient care across medical specialities and varied service providers.

Several studies found strong evidence on the association between lifetime traumas and increased utilization of primary health care. The household survey in England and Wales found that adults with 4 ACEs were twice as likely to visit a general practitioner 6 times or more in the last 12 months (OR 2.3, 95% CI 1.8-2.9) compared to adults with no ACEs.[9] The Australian Longitudinal Study of Women's Health found that women with lifetime violence experiences had almost twice the odds of higher general practice service use (AOR 1.82, 95% CI 1.37–2.40), compared with women without any violence experiences.[10] A majority of patients in community mental health and substance abuse services experienced repeated trauma throughout their life span.[11] Therefore, primary health care services must be designed in a way that will support the recovery of survivors. However, they often have the opposite effect and can trigger memories about traumatic experiences

For peer review only - http://bmjopen.bmj.com/site/about/guidelines.xhtml

through invasive procedures and coercive practices (e.g., the removal of choice regarding treatment or judgmental attitudes following a disclosure of abuse; lack of available and acceptable services).[12] Re-activation of traumatic experiences within health services can affect both service users and staff, with the latter experiencing vicarious trauma.[13]

The field of trauma-informed (TI) approach to care (synonyms *TI care*, *TI practice, TI model of care, TI service system*) is relatively new. The concept of TI approach was developed in the US,[14] where it is widely used across all sectors.[1] Various organisations, expert panels, and researchers proposed varied definitions of TI care. One of the consensus-based definitions describes TI care as "a strengths-based service delivery approach that is grounded in an understanding of and responsiveness to the impact of trauma, that emphasizes physical, psychological, and emotional safety for both providers and survivors, and that creates opportunities for survivors to rebuild a sense of control and empowerment".[15] Through consultations with the expert panel, SAMHSA developed a flexible framework comprising foundation assumptions, principles and implementation domains for a TI care[1] that can be adapted to any service system including primary health care.[16] SAMHSA's framework of TI approach is based on the four key assumptions of:

1. Recognition. All people in an organisation recognise how patients' and staff's experiences of trauma might affect the way they think, feel and behave.
2. Realisation. All staff in the organisation accept how trauma can affect people, and patient's behaviour is understood in the context of coping with their experiences.
3. Response. The organisation acts to effectively integrate knowledge about prevalence and impact of trauma into policies, procedures, and practices.

For peer review only - http://bmjopen.bmj.com/site/about/guidelines.xhtml

4. Resist the re-activation of traumatic memories. Steps are taken to prevent further traumatising both service users and staff through a focus on the recovery of survivors, as well as the wellbeing of staff.[1]

SAMHSA's TI approach framework includes six key principles applicable to varied settings: (i) safety, (ii) trustworthiness and transparency, (iii) peer support, (iv) collaboration and mutuality, (v) empowerment, choice and choice, and (vi) cultural/historical/gender issues.

A TI approach is distinct from trauma-specific interventions (e.g., trauma-focused cognitive behavioural therapy) or trauma services (e.g., Traumatic Stress Service) that treat trauma symptoms. A TI approach can include trauma-specific interventions, although the essential component is the application of the above assumptions and principles in the organisational/system levels.[1]

Most extant evidence for TI approaches comes from Northern America.[17-19] TI care has only recently been included in the UK National Health Service (NHS) long-term plan[20] and the NHS mental health implementation plan.[21] The Scottish Government[22] and the Safeguarding Board for Northern Ireland[23] endorsed TI approaches across health care, social care, education, and justice sectors. Public Health Wales published reports recommending TI approaches across public services.[24] TI approaches were endorsed in local governments policies across England.[25] However, the evidence base for TI approaches to health care is not well established. A recent scoping review identified only a few examples of TI care implemented in Scotland[22] and England[26,27] and recommended developing the evidence base to demonstrate the value of TI approaches in the UK healthcare context.[28]

For peer review only - http://bmjopen.bmj.com/site/about/guidelines.xhtml

The aim of this systematic review is to synthesize evidence on TI approaches in primary care and community mental health care globally, which will help inform the development of a UK specific model of TI in these settings. The synthesis will address the following research questions:

1. What models of TI care have been used in primary care and community mental health services?
2. What are the formal theories and empirically supported theories of change underpinning these models and their evaluations?
3. What evidence is available for the acceptability, effectiveness, and cost-effectiveness of TI approaches to primary care and community mental health care?

Methods and analysis

This protocol follows the Preferred Reporting Items for Systematic Review and Meta-Analysis Protocol (PRISMA-P) checklist (Appendix 1).[29] The systematic review will be conducted and reported following the PRISMA guidelines.[30]

Eligibility criteria

We will select studies according to the following criteria.

Population

Patients in primary and community mental health care (globally) aged 18 and over. Professionals who plan, commission, and deliver primary or community mental health care.

Intervention

We will use the SAMHSA definitions of TI approach[1] and include evaluations of any model of TI care in primary or community mental health organisations. The SAMHSA

For peer review only - http://bmjopen.bmj.com/site/about/guidelines.xhtml

framework of TI care covers ten implementation domains of organisational change in (1) governance and leadership, (2) written policies and protocols, (3) physical environment, (4) training and workforce development, (5) engagement and involvement of service users, (6) cross-sector collaboration, (7) progress monitoring and quality assurance, (8) financing, (9) evaluation, (10) screening, assessment, and treatment for trauma.

We will exclude studies of trauma-specific interventions that treat trauma symptoms.

Comparator

Primary and community mental health care not using TI approaches.

Outcomes

We identified outcomes from the prior literature on TI approaches to health care[17, 31-34] and consultations with two study advisory groups of trauma survivors and professionals who plan, commission and deliver primary and community mental health care. To map the outcomes from all these sources on the draft logic model of a TI care, we adapted a published measurement model for TI primary care (Figure 1).[34] Throughout review process, we will refine this draft logic model in discussions within the team and consultations with the study advisory groups.

For peer review only - http://bmjopen.bmj.com/site/about/guidelines.xhtml

To be included, a study must report a measure from at least one of the above outcome categories. The TI approach is an organisation level intervention. Therefore, we define outcomes at the organisation level as main outcomes and outcomes at the individual level as additional outcomes. We will also look at formal theories and empirically supported theories of change underpinning included TI approaches and their evaluations. We will pay special attention to adverse effects of TI approaches on patient health, health care providers (e.g., vicarious trauma), service utilisation and quality of care.

Types of studies

Primary studies of any design that evaluated the acceptability and/or effectiveness and/or cost-effectiveness of TI approaches in primary and/or community mental health care will be included. We will include studies with mixed samples only if outcomes for the primary healthcare and/or community mental healthcare sub-sample are reported separately irrespective of the proportion of the sub-sample. Reference lists of systematic reviews that meet this criterion will be searched to identify relevant primary studies. Editorials, policy documents, and books will be excluded.

Setting

Any setting providing primary care, including primary care mental health services. WHO defines a primary health care centre as setting providing services that are usually the first point of contact with a health care professional.[35] Depending on the country, they can include any open access, community based first point of care service, for example, general practice clinics, community-based units, basic health units, family health strategy, primary care home visits, day-care centres, multicentre

For peer review only - http://bmjopen.bmj.com/site/about/guidelines.xhtml

health clinics, one stop crisis centre, Improving Access to Psychological Therapies (IAPT) services.[8]

Time frame

An early and influential paper[14] discussing TI approaches was published in 2001. However, 1990 was chosen as a starting point to capture any relevant and early discussions of trauma-informed care principles from a global perspective. We will limit the studies by date to ensure that the search identifies all relevant studies since this publication.

Language

There will be no language restrictions, provided an English language abstract is available for initial screening. During full-text screening, if the included papers are not available in English, we will translate them with help from multilingual colleagues and Google Translate.

Search strategy

SD and NVL will develop a comprehensive search strategy using a combination of MeSH and free-text terms, based on previous systematic reviews in the areas of TI care[32,36] and the expertise of the research team to identify relevant papers on TI approaches in primary and community mental health care for adults. SD will run several scoping exercises in different electronic databases to maximise the sensitivity and specificity of the developed search strategy (Appendix 2, example search strategy).

SD will search electronic bibliographic databases for potential primary studies from January 1990- February 2020: Cochrane Library, MEDLINE, EMBASE, Cumulative

For peer review only - http://bmjopen.bmj.com/site/about/guidelines.xhtml

Index to Nursing and Allied Health Literature (CINAHL EBSCO) and PsycINFO and update searches in the last two months of the study.

In addition, SD will search the PROSPERO database to identify any relevant systematic reviews in progress. She will also conduct a grey literature search to identify studies not indexed in the databases listed above. SD will search websites of organisations involved in development and implementation of TI care: UK national and local governments, King's Fund, SAMHSA, Violence, Abuse and Mental Health Network and Trello (Adverse Childhood Experiences Resource library). Theses and dissertations will be identified through ethos library and PROQUEST. SD, AB, NVL will carry out forward and backward referencing of included papers to supplement the database and grey literature searches to identify any further relevant articles. SD and NVL will approach corresponding authors of included papers, study advisory groups, and experts in the field of trauma and primary care for additional relevant articles.

Screening of studies

References will be managed in Rayyan (https://rayyan.qcri.org/). SD will export search results from the different databases into the Rayyan database and remove duplicates. Study selection will be completed in two stages: first, titles and abstracts will be screened; next full-text will be screened to identify studies eligible for inclusion. Two members of the research team (SD and AB or NVL) will independently screen titles, abstracts and full-text. Any discrepancies between reviewers will be discussed with other team members.

We will list excluded full text studies in the table categorised by reasons for exclusion. We will collate multiple reports of the same study so that each study, rather than each report, is the unit of analysis in the review. We will also provide any

For peer review only - http://bmjopen.bmj.com/site/about/guidelines.xhtml

information we can obtain from corresponding authors about ongoing studies. We will record the screening process in detail to complete a PRISMA flow diagram.[37]

Data extraction

A data extraction form will be used to focus on the characteristics that are relevant to this review:

1. Methods: type of study (randomised trial, interrupted time series, controlled/uncontrolled before-after, cross-sectional, qualitative, mixed-method, service evaluation).
2. Study setting (country, key features of the health care system, health care setting)
3. Characteristics of the participants (age, sex, ethnicity, condition as described and identified by the authors of included studies)
4. Characteristics of the TI approach: components, comparison. We will map components of each TI model on the SAMHSA's ten-domains framework of organisational change[1]
5. Outcomes: main and additional outcomes specified and collected, time points reported
6. Theories underpinning the TI approach: formal theories and/or empirically developed theories of change explaining how the intervention works. We will also extract data on theories underpinning included evaluations.

We will seek input from the study advisory groups of trauma survivors and professionals on any other relevant data that should be extracted. SD will pilot the adapted extraction form on publications of a quantitative, qualitative and mixed-method study, and then refine it. To minimise bias and errors, one reviewer (SD) will extract the data and a second reviewer (AB or NVL) will check the extraction in

For peer review only - http://bmjopen.bmj.com/site/about/guidelines.xhtml

detail. Any disagreements between reviewers will be resolved through discussions and, if required, with other team members. We will ask corresponding authors of included studies to check reconciled data extraction forms and provide missing information and clarifications.

Quality appraisal

We will use the Mixed Methods Appraisal Tool (MMAT) tool to appraise the studies[38] Quality appraisal will be carried out as part of data extraction. SD will complete MMAT checklists for each study. Second reviewer (AB or NVL) will check completed checklists in detail. Any disagreement between the reviewers will be resolved through discussions and if required by other members of the team.

Data Synthesis

Synthesis of quantitative data. Based on feasibility searches and background reading, we expect that included quantitative studies will report outcomes that vary substantially by the way they were defined and measured. For this reason, we anticipate that a quantitative synthesis of the results from the quantitative studies will not be appropriate. We will, therefore, employ narrative synthesis to summarise findings from quantitative studies. This will involve the use of descriptive text and tables to summarise data to allow readers to consider findings in the light of differences in study designs. We will describe all TI models in a table based on the TiDIER template.[39] For each model of TI care, we will describe the range of effects found in the studies and if possible, the theory of change through which the TI models were intended to affect specific outcomes.

Synthesis of qualitative data. For qualitative studies, we plan to use the thematic synthesis method.[40]

For peer review only

For peer review only - http://bmjopen.bmj.com/site/about/guidelines.xhtml

Synthesis of quantitative and qualitative findings. We will use a results-based convergent synthesis design,[41] following the Sandelowski's segregated method.[42] First, we will analyse and synthesise the quantitative (narrative synthesis) evidence and the qualitative (thematic synthesis) separately as described above. Next, we will then integrate the synthesis products (results of both syntheses) using another method of synthesis (e.g., tables, matrices or reanalysing evidence as a result of both syntheses), which allows for comparing and/or juxtaposing the findings from the quantitative and qualitative evidence.[41] At this stage, we will map all the evidence on the refined logic model of TI care (Figure 1) and finalise it through discussions within the team and consultations with the survivor and professional advisory groups.[43]

Discussion

An effective response to (often) hidden trauma of patients in general practice and primary care mental health services is long overdue. This is a protocol for a systematic review on TI approaches in primary care and community mental health care that addresses the gap in evidence on the acceptability, effectiveness, and cost-effectiveness of TI health care. The study includes both peer-reviewed and grey literature and offers a global view of TI approaches. Our findings will inform the development of an evidence-based UK-specific model of TI primary care and community mental health care. Although the output of this review will form the basis for further research, the findings will be relevant to current policy and practice, even before we have developed and tested the UK-specific TI model. UK policymakers can use this new evidence when developing/amending health policies on TI care. Involvement of two advisory groups of trauma survivors and providers of health care

For peer review only - http://bmjopen.bmj.com/site/about/guidelines.xhtml

throughout all stages of this review helps to produce evidence that is relevant to end-users and is likely to be translated into policy and practice.

A limitation of this review is the use of search terms based on the current TI terminology, which was introduced in early 2000 (Appendix 2). We might miss the earlier studies which evaluated health care services with TI approaches that were not labelled as such. We addressed this limitation by designing a search strategy with input from our advisory groups. We included a term for psychologically informed environments from the pre-TI era and also undertook searches from 1990. Another limitation of the review is that it will exclude studies without an abstract in the English language, which could lead to missing relevant studies. Policy documents will be excluded from this review and is an area for a future policy review.

This study was conceived and designed at the pre-COVID-19 pandemic era and will be delivered and disseminated throughout and after the pandemic. The WHO[44] and statutory and third sector organisations[45,46] have already reported that stress, social isolation measures, quarantines at home, etc. resulted in the increase of all forms of family violence. Consultation with our advisory groups on the impact of the pandemic has supported these findings. Our lay and professional contributors talked about the rise in traumatic experiences among patients and health care professionals, worsening mental health, increased demand for health and social services and transition of services from face-to-face delivery to phone or online delivery mode. This changing environment makes our systematic review of TI approaches to health care timely in providing evidence for effective and acceptable primary and community mental health care in the post-pandemic era.

For peer review only - http://bmjopen.bmj.com/site/about/guidelines.xhtml

Patient and Public Involvement

We have set up two advisory groups to work with researchers throughout the study: a trauma survivor group and a professional group. The trauma survivor advisory group consists of eight people with diverse lived experiences of trauma who had been recipients of care in the NHS and other care systems. The professional advisory group consists of eight professionals (e.g., planning and development manager from the local authority, clinical psychologist, clinical lead specialist) from England and Wales who are involved in planning, funding, commissioning, or delivering primary health care or community mental health services.

We organised separate meetings with the survivor and professional advisers where they were introduced to the study and systematic review process. Both groups took part in brainstorming exercises on formulating research questions and listing outcomes for the systematic review that are meaningful to patients, practitioners, service managers and commissioners. Based on their feedback, SD and NL mapped the outcomes to those identified in the existing literature independently, and any discrepancies were resolved through discussions. The professional advisory group developed a list of UK primary and community mental health services. The group highlighted inconsistent terminology used in the UK (i.e., ACEs, TI approaches/care/practice, psychologically informed environments) and diverse approaches to developing policies on TI care across the UK (i.e., top-down in Scotland, Northern Ireland, and Wales vs bottom-up in England). They highlighted the need for synthesised research evidence about such approaches that are relevant to the UK and for using this evidence to inform health policy. We will meet with the survivor and professional advisory groups biannually to consult on data extraction, interpretation, and dissemination of study findings.

For peer review only - http://bmjopen.bmj.com/site/about/guidelines.xhtml

Ethics and dissemination

No ethical approval is required for this systematic review, as this does not involve the collection of primary data. Findings of this review will be disseminated through publication of a peer-reviewed paper, papers presented at conferences for academic and practitioner audiences, through local clinical commissioning groups and policy briefings targeted at local and national policymakers. Our survivor and professional advisers will be consulted for sources through which we should disseminate the findings. We will also work with survivor advisers to develop a lay summary of the study findings which will be disseminated through the Centre for Academic Primary Care PPI contributors. Finally, we will produce a policy brief of the study findings for dissemination among professional stakeholders involved in planning, funding, commissioning and delivery of primary and community mental health care.

Figure legend

Figure 1: Draft logic model of trauma-informed primary and community mental health care. Adapted from German et al. (2020)[34]

Acknowledgements

We would like to thank Mike Bell (BRC PPI coordinator) for supporting us with the set up and coordination of study advisory groups and our survivor and professional advisory group for their contribution in identifying outcomes for this systematic review.

Author contributions

NVL conceived the idea and secured funding. NVL and SD designed the study. SD drafted the study protocol. SD, AB and NVL set up and coordinate study advisory

For peer review only - http://bmjopen.bmj.com/site/about/guidelines.xhtml

groups. SD and NVL produced first draft of the manuscript. All co-authors (SD, AB, GF, JM, KT, SZ, NVL) contributed to the refinement of the manuscript and approved the final version for submission.

Funding statement

This study is funded by the NIHR Biomedical Research Centre at University Hospitals Bristol NHS Foundation Trust and the University of Bristol. Grant reference number R100514-105. The views expressed are those of the authors and not necessarily those of the NIHR or the Department of Health and Social Care.

Competing interest statement

Authors have no conflict of interest to declare.

For peer review only

References

1. Substance Abuse and Mental Health Services Administration (SAMHSA). *SAMHSA's concept of trauma and guidance for a trauma-informed approach*. SAMHSA; 2017. https://store.samhsa.gov/system/files/sma14-4884.pdf p. 27, Accessed 10th February 2020.
2. Kessler RC et al. Trauma and PTSD in the WHO World Mental Health Surveys. *Eur J Psychotraumatol*, 2017;**8**:5; p. 1353383.
3. Bellis MA et al. National household survey of adverse childhood experiences and their relationship with resilience to health-harming behaviors in England. *BMC Med.* 2014;**12**: p. 72.
4. Office for National Statistics. *Domestic abuse in England and Wales: year ending March 2018*; 2018. https://www.ons.gov.uk/peoplepopulationandcommunity/crimeandjustice/bulletins/domesticabuseinenglandandwales/yearendingmarch2018#prevalence-of-domestic-abuse, Accessed 10th February 2020.
5. Magruder KM, McLaughlin KA et al. Trauma is a public health issue. *Eur J Psychotraumatol*, 2017; **8**(1): p. 1375338.
6. World Health Organization. Declaration of alma-ata: World Health Organization, Regional Office for Europe: WHO, 2004.
7. World Health Organisation. Integrating mental health services into primary health care. Geneva: WHO, 2007 (http://www.who.int/mental_health/policy/services/en/index.html, Mental Health Policy, Planning and Service Development Information Sheet, Sheet3). Accessed 9th May 2020.

For peer review only - http://bmjopen.bmj.com/site/about/guidelines.xhtml

8. Charles A, Ham C et al. Reimagining community services. Making the most of our assets. The King's Fund, 2018, https://www.kingsfund.org.uk/publications/community-services-assets. p. 118, Accessed 4th May 2020.
9. Bellis M, Hughes K et al. The impact of adverse childhood experiences on health service use across the life course using a retrospective cohort study. *J of Health Serv Res & Pol*. 2017; **22**(3): p. 168-177.
10. Coles J, Lee A. et al. General practice service use and satisfaction among female survivors of childhood sexual abuse. *Aust Fam Physician*. 2015; **44**(1): p. 71-76.
11. Rosenberg L. Addressing Trauma in Mental Health and Substance Use Treatment. *Journal of Beh Health Serv & Res*. 2011; **38**(4): p. 428-431.
12. Department of Health & Social Care. The Women's Mental Health Taskforce: Final report. London; 2018. Available from: https://assets.publishing.service.gov.uk/government/uploads/system/uploads/attachment_data/file/765821/The_Womens_Mental_Health_Taskforce_-_final_report1.pdf, Accessed 4th January 2021.
13. Figley CR, Kleber RJ. *Beyond the "Victim"*. In: Kleber R.J., Figley C.R., Gersons B.P.R. (eds) Beyond Trauma. The Plenum Series on Stress and Coping. 1995; Springer, Boston, MA.
14. Harris M and Fallot R, *Using trauma theory to design service systems*. 2001, San Francisco, CA: Jossey-Bass.
15. Hopper EK, Bassuk EL, Olivet J: Shelter from the storm: trauma-informed care in homelessness services setting. *The Open Health Services and Policy Journal*. 2010; 3: 80-100.

For peer review only - http://bmjopen.bmj.com/site/about/guidelines.xhtml

16. Gerber MR. Trauma-informed healthcare approaches. 2019, Cham, Switzerland: Springer Nature.

17. Melz H, Morrison C et al. Review of trauma-informed initiatives at the systems level. Trauma-informed approaches: connecting research, policy, and practice to build resilience in children and families. 2019. https://aspe.hhs.gov/system/files/pdf/262051/TI_Approaches_Research_Review.pdf. p.37, Accessed 18th January 2020.

18. Raja S, Hasnain M et al. Trauma informed care in medicine: current knowledge and future research directions. *Fam Community Health*. 2015; **38**(3):216-226.

19. Reeves E. A synthesis of the literature on trauma-informed care. *Issues Ment Health Nurs*. 2015. **36**(9): 698-709.

20. NHS. The NHS Long Term Plan. 2019. https://www.longtermplan.nhs.uk/wp-content/uploads/2019/08/nhs-long-term-plan-version-1.2.pdf, Accessed 20th January 2020.

21. NHS. Mental Health Implementation Plan 2019/20 – 2023/24. 2019. https://www.longtermplan.nhs.uk/wp-content/uploads/2019/07/nhs-mental-health-implementation-plan-2019-20-2023-24.pdf, Accessed 20th January 2020.

22. NHS Education for Scotland. Transforming psychological trauma: a knowledge and skills framework for the Scottish workforce. 2017. https://www.nes.scot.nhs.uk/media/3971582/nationaltraumatrainingframework.pdf Accessed 20th January 2020.

23. Bunting L, Montgomery L et al., Developing trauma informed practice in Northern Ireland: Key messages. 2019, Queen's University, Belfast: https://pureadmin.qub.ac.uk/ws/portalfiles/portal/168356931/ACEs_Report_A4_Feb_2019_Key_Messages.pdf p. 33, Accessed 20th January 2020.

For peer review only - http://bmjopen.bmj.com/site/about/guidelines.xhtml

24. Public Health Wales NHS Trust. Adverse Childhood Experiences and their association with chronic disease and health service use in the Welsh adult population. Walsh Adverse Childhood Experiences (ACE) study. 2016. http://www.wales.nhs.uk/sitesplus/documents/888/ACE%20Chronic%20Disease%20report%20%289%29%20%282%29.pdf, Accessed 25th May 2020.

25. Greater London Authority. Connecting up the care. Supporting London's children exposed to domestic abuse, parental mental ill-health and parental substance abuse. 2020. https://www.london.gov.uk/sites/default/files/connecting_up_the_care.pdf, Accessed 25th May 2020.

26. Trauma-informed care. 2019. https://www.tewv.nhs.uk/services/trauma-informed-care/, Accessed 28th January 2020.

27. Petrillo M et al. Healing Trauma evaluation report. 2019: https://researchportal.port.ac.uk/portal/files/14351866/HT_evaluation_full_report_June_2019.pdf, Accessed 28th January 2020.

28. Sweeney A, Clement S et al. Trauma-informed mental healthcare in the UK: what is it and how can we further its development? *Mental Health Review Journal*. 2016; **21**(3): p. 174-192.

29. Shamseer L, Moher D, Clarke M et al. Preferred reporting items for systematic review and meta-analysis protocols (PRISMA-P) 2015: elaboration and explanation. *BMJ* 2015; **350**: g7647.

30. Moher D, Liberati A, Tetzlaff J et al. Preferred reporting items for systematic reviews and meta-analyses: the PRISMA statement. *BMJ* 2009; **339**: b2535.

For peer review only - http://bmjopen.bmj.com/site/about/guidelines.xhtml

31. The Scottish Government. Survivor Scotland. Strategic outcomes and priorities 2015-17. 2015. https://www.gov.scot/publications/survivorscotland-strategic-outcomes-priorities-2015-2017/pages/1/, Accessed 21st February 2020.

32. Champine RB, Lang JM et al. Systems Measures of a Trauma-Informed Approach: A Systematic Review. *Am J Community Psychol.* 2019; **64**(3): 418-437.

33. Robotham D, Sweeney A et al. Violence, Abuse and Mental Health Network. Survivors' priority themes and questions for research. *Consultation Report*. 2019, Violence, Abuse and Mental Health Network: https://www.vamhn.co.uk/uploads/1/2/2/7/122741688/consultation_report_on_website.pdf, Accessed 21st February 2020.

34. Germán M et al. Measuring the impact of trauma-informed primary care: are we missing the forest for the trees? 2020, Montefiore Medical Group: https://www.chcs.org/resource/measuring-the-impact-of-trauma-informed-primary-care-are-we-missing-the-forest-for-the-trees/. p.15, Accessed 12th May 2020.

35. World Health Organisation. A vision for primary health care in the 21st century: towards universal health coverage and the Sustainable Development Goals: Geneva: WHO, 2018. https://www.who.int/docs/default-source/primary-health/vision.pdf, Accessed 12th May 2020.

36. Branson CE, Baetz CL et al. Trauma-Informed Juvenile Justice Systems: A Systematic Review of Definitions and Core Components. *Psychol Trauma:* 2017; **9**(6): 635-646.

For peer review only - http://bmjopen.bmj.com/site/about/guidelines.xhtml

37. Liberati A, Altman DG et al. The PRISMA statement for reporting systematic reviews and meta-analyses of studies that evaluate health care interventions: explanation and elaboration. *BMJ.* 2009; **339**: b2700.

38. Hong QN, Pluye P et al. Mixed Methods Appraisal Tool (MMAT), version 2018, Registration of Copyright (#1148552), Canadian Intellectual Property Office, Industry Canada. http://mixedmethodsappraisaltoolpublic.pbworks.com/w/page/127425845/Download%20the%20MMAT, Accessed 4th January 2021.

39. Hoffmann TC, Glasziou PP et al. Better reporting of interventions: template for intervention description and replication (TIDieR) checklist and guide. *BMJ*. 2014; **348**:1687.

40. Thomas J. and Harden A. Methods for the thematic synthesis of qualitative research in systematic reviews. *BMC Med Res Methodol*. 2008; **8**: 45.

41. Hong QN, Pluye P et al. Convergent and sequential synthesis designs: implications for conducting and reporting systematic reviews of qualitative and quantitative evidence. *Syst Rev. 2017;* **6** (1): 61.

42. Sandelowski M, Voils CI, Barroso J. Defining and designing mixed research synthesis studies. *Res Schools*. 2006;13(1):29–40.

43. Rohwer A, Pfadenhauer L et al. Series: Clinical Epidemiology in South Africa. Paper 3: Logic models help make sense of complexity in systematic reviews and health technology assessments. *J Clin Epidemiol.* 2017; 83:37-47.

44. World Health Organisation. COVID-19 and violence against women. What the health sector/system can do. WHO, 2020. https://www.who.int/reproductivehealth/publications/VAW-COVID19-Rev1.pdf. p. 3, Accessed 25th May 2020.

For peer review only - http://bmjopen.bmj.com/site/about/guidelines.xhtml

45. Women's Aid. The impact of COVID-19 on women and children experiencing domestic abuse, and the life-saving services that support them. 2020. https://www.womensaid.org.uk/the-impact-of-covid-19-on-women-and-children-experiencing-domestic-abuse-and-the-life-saving-services-that-support-them/, Accessed 13th April 2020.

46. UNICEF. The Alliance for Child Protection in Humanitarian Action, Technical Note: Protection of Children during the Coronavirus Pandemic. 2020. https://www.unicef.org/documents/technical-note-protection-children-coronavirus-disease-2019-covid-19-pandemic, Accessed 30th May 2020.

For peer review only

For peer review only - http://bmjopen.bmj.com/site/about/guidelines.xhtml

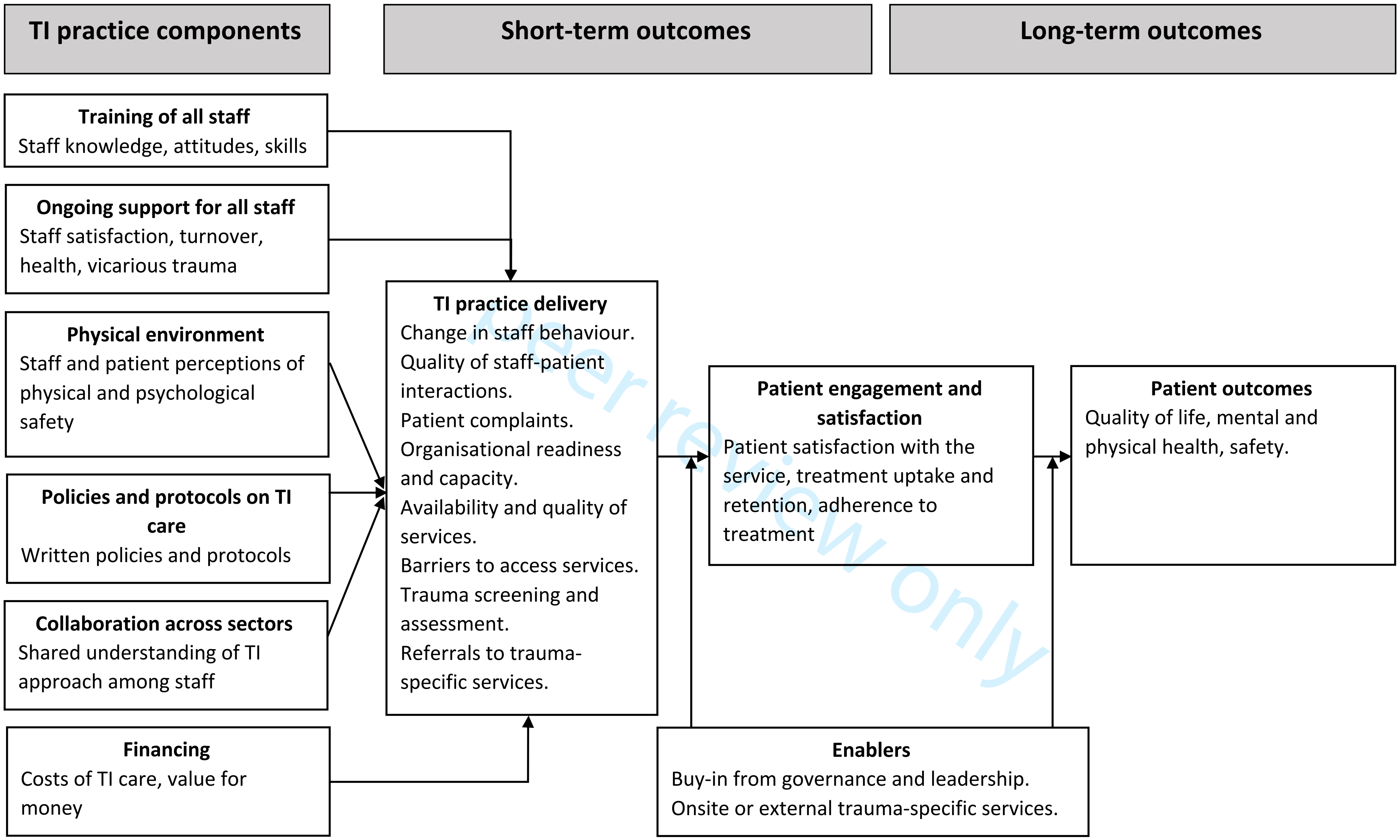

Figure 1: Draft logic model of trauma-informed primary and community mental health care. Adapted from German et al. (2020)[34]

peer review only

Appendix 1: Preferred Reporting Items for Systematic review and Meta-Analysis Protocols (PRISMA-P) checklist (Shamseer et al. 2015)[29]

Section and topic	Item No	Checklist item	Reported on Page #
**Administrative Information**			
**Title**			
Identification	1a	Identify the report as a protocol of a systematic review	1 (Title)
Update	1b	If the protocol is for an update of a previous systematic review, identify as such	NA
Registration	2	If registered, provide the name of the registry (e.g., PROSPERO) and registration number in the Abstract	4 (Abstract)
**Authors**			
Contact	3a	Provide name, institutional affiliation, and e-mail address of all protocol authors; provide physical mailing address of corresponding author	1-2
Contributions	3b	Describe contributions of protocol authors and identify the guarantor of the review	19-20 (Author Contributions)
Amendments	4	If the protocol represents an amendment of a previously completed or published protocol, identify as such and list changes; otherwise, state plan for documenting important protocol amendments	NA
**Support**			
Sources	5a	Indicate sources of financial or other support for the review	20 (Funding statement)
Sponsor	5b	Provide name for the review funder and/or sponsor	20 (Funding statement)
Role of sponsor/funder	5c	Describe roles of funder(s), sponsor(s), and/or institution(s), if any, in developing the protocol	NA
**Introduction**			
Rationale	6	Describe the rationale for the review in the context of what is already known	5-8 (Introduction)
Objectives	7	Provide an explicit statement of the question(s) the review will address with reference to participants, interventions, comparators, and outcomes (PICO)	9, 9-12 (PICO)
**Methods**			
Eligibility criteria	8	Specify the study characteristics (e.g., PICO, study design, setting, time frame) and report characteristics (e.g., years considered, language, publication status) to be used as criteria for eligibility for the review	9-12 (eligibility criteria)

For peer review only - http://bmjopen.bmj.com/site/about/guidelines.xhtml

Information sources	9	Describe all intended information sources (e.g., electronic databases, contact with study authors, trial registers, or other grey literature sources) with planned dates of coverage	12-13 (search strategy)
Search strategy	10	Present draft of search strategy to be used for at least one electronic database, including planned limits, such that it could be repeated	Appendix 2 (Supplementary file)
Study records			
Data management	11a	Describe the mechanism(s) that will be used to manage records and data throughout the review	13 (Screening of studies, paragraph 1)
Selection process	11b	State the process that will be used for selecting studies (e.g., two independent reviewers) through each phase of the review (i.e., screening, eligibility, and inclusion in meta-analysis)	13 (Screening of studies, paragraph 1)<br>14 (Data extraction, paragraph 2)
Data collection process	11c	Describe planned method of extracting data from reports (e.g., piloting forms, done independently, in duplicate), any processes for obtaining and confirming data from investigators	14 (Data extraction, paragraph 2)
Data items	12	List and define all variables for which data will be sought (e.g., PICO items, funding sources), any pre-planned data assumptions and simplifications	14 and 15 (Data extraction)
Outcomes and prioritization	13	List and define all outcomes for which data will be sought, including prioritization of main and additional outcomes, with rationale	Figure 1 (Supplementary file)
Risk of bias in individual studies	14	Describe anticipated methods for assessing risk of bias of individual studies, including whether this will be done at the outcome or study level, or both; state how this information will be used in data synthesis	15 (Quality appraisal)
Data Synthesis	15a	Describe criteria under which study data will be quantitatively synthesized	NA
	15b	If data are appropriate for quantitative synthesis, describe planned summary measures, methods of handling data, and methods of combining data from studies, including any planned exploration of consistency (e.g., I^2, Kendall's tau)	NA
	15c	Describe any proposed additional analyses (e.g., sensitivity or subgroup analyses, meta-regression)	NA
	15d	If quantitative synthesis is not appropriate, describe the type of summary planned	15 (Synthesis)
Meta-bias(es)	16	Specify any planned assessment of meta-bias(es) (e.g., publication bias across studies, selective reporting within studies)	NA
Confidence in cumulative evidence	17	Describe how the strength of the body of evidence will be assessed (e.g., GRADE)	NA

*NA- Not Applicable

For peer review only

Appendix 2: Example search strategy

Database(s): **Ovid MEDLINE(R)** 1946 to present

Search Strategy:

#	Searches	Results
1	(trauma inform* or trauma-inform* or trauma focus* or trauma-focus* or trauma responsive* or trauma sensitive or trauma-sensitive or trauma services or complex trauma or trauma-base* or trauma base*).ab,ti.	2648
2	(trauma-inform* and (approach* or care or practi? or intervention* or system*)).ab,ti.	754
3	(trauma-inform* and (measure or survey or questionnaire or checklist or assessment or evaluation)).ab,ti.	279
4	or/1-3	2648
5	(adverse childhood event$ or adverse childhood experience$ or psychological* informed environment$).ab,ti.	1595
6	4 or 5	4152
7	(child or child* or adolescent*).mp.	2378418
8	child/ or infant/ or adolescent/	3091467
9	or/7-8	3568141
10	6 not 9	1468

only
